# Supplementary material for: Neuromodulatory connectivity defines the structure of a behavioral neural network
Source: eLife. 2017 Nov 22;6:e29797. doi: 10.7554/eLife.29797 (PMC5720592; doi:10.7554/eLife.29797)
Supplement: Supplementary file 1. [file elife-29797-supp1.docx]

**Supplementary File 1: Fly Genotypes Used Listed by Figure**

**Figure 1A:** w; CCAP-GAL4DBD^K2A2^; ETHRA^MI00949^-p65AD/ 20XUAS-6XEGFP

**Figure 1B:**

Control (Top): w; UAS-Kir2.1/ CCAP-GAL4DBD^K2A2^; UAS-Kir2.1

Experimental (Bottom): w; UAS-Kir2.1/ CCAP-GAL4DBD^K2A2^; ETHRA^MI00949^-p65AD/ UAS-Kir2.1

**Figure 1C:**

Control (Top): w; UAS-dTrpA1/ CCAP-GAL4DBD^K2A2^; +

Experimental (Bottom): w; UAS-dTrpA1/CCAP-GAL4DBD^K2A2^ ; ETHRA^MI00949^-p65AD/+

**Figure 2A:**

Left: w;; Rk^pan^-GAL4/20XUAS-6XEGFP

Middle: w;; CCAP-R^MI05804^-GAL4/20XUAS-6XEGFP

Right: w; Rk^TGEM^-GAL4DBD; CCAP-R^MI05804^-p65AD/20XUAS-6XEGFP

**Figure 2B & C:**

Rk-Gal4: w; UAS-Kir2.1/tubP-Gal80^ts^-20; Rk^pan^-GAL4/ UAS-Kir2.1

w^1118^: w^1118^; UAS-Kir2.1/tubP-Gal80^ts^-20; UAS-Kir2.1

CCAP-R-Gal4: w;UAS-Kir2.1/tubP-Gal80^ts^-20; CCAP-R^MI05804^-GAL4/ UAS-Kir2.1

**Figure 3A:** w; VGlut^MI04979^ -Gal4DBD/Rk^TGEM^-p65AD; 20XUAS-6XEGFP

**Figure 3A’:** w; Rk^TGEM^-Gal4DBD; ETHRA^MI00949^-p65AD/20XUAS-6XmCherry

**Figure 3A”:** w; Rk^TGEM^-Gal4DBD; ETHRB-p65AD/20XUAS-6XmCherry

**Figure 3B & C:** Experimental : w; Burs-LexA::VP16AD /LexA_op_-P2X_2_; Rk^pan^-Gal4/ UAS-GCaMP6s

Control: w;+/LexA_op_-P2X_2_; Rk^pan^-Gal4/ UAS-GCaMP6s

**Figure 4A & C (top):** w; UAS-GCaMP6s/+; Rk^pan^-Gal4/ UAS-GCaMP6s

**Figure 4B & C (bottom):** w; UAS-GCaMP6s/+; 24B-Gal4/ UAS-GCaMP6s

**Figure 5A-C:** w; UAS-GCaMP6s/+; Rk^pan^-Gal4/ UAS-GCaMP6s

**Figure 5D-F:** w; UAS-GCaMP6s/+; 24B-Gal4/ UAS-GCaMP6s

**Figure 5G:** Experimental w; VGlut ^MI04979^-LexA::QFAD/LexA_op_-GCaMP3.0; Rk^pan^-Gal4/ UAS-P2X_2_

Control: w; VGlut ^MI04979^-LexA::QFAD/LexA_op_-GCaMP3.0; +/ Rk^pan^-Gal4

**Figure 6A:** w; +/VGlut ^MI04979^-Gal4DBD; CCAP-R^MI05804^-p65AD/20XUAS-6XEGFP

**Figure 6B-C, D (bottom) &E:** w; VGlut ^MI04979^-Gal4DBD/UAS-GCaMP6s; CCAP-R^MI05804^-p65AD/UAS-GCaMP6s

**Figure 6D (top) & E:** w; UAS-GCaMP6s; Rk^pan^-Gal4/ UAS-GCaMP6s

**Figure 6F:** Experimental : w; Rk^TGEM^-LexA::QFAD/ LexA_op_-P2X_2_; CCAP-R^MI05804^-Gal4/ UAS-GCaMP6s

Control: w; +/LexA_op_-P2X_2_; CCAP-R^MI05804^-Gal4/ UAS-GCaMP6s

**Figure 7A:** w;; ETHRB-Gal4/20XUAS-6XEGFP

**Figure 7B:** w;CCAP-Gal80; ETHRA-Gal4/20XUAS-6XEGFP

**Figure 7C:**

Control (top): w; UAS-Kir2.1/tubP-Gal80^ts^-20; UAS-Kir2.1/+

Experimental (bottom): w; UAS-Kir2.1/tubP-Gal80^ts^-20; ETHRB-GAL4/ UAS-Kir2.1

**Figure 7 D:**

Control (top): w^1118^; UAS-Kir2.1; UAS-Kir2.1

Experimental (bottom): w; UAS-Kir2.1/CCAP-Gal80; tubP-Gal80^ts^-20/ ETHRA-GAL4/ UAS-Kir2.1

**Figure 6—Figure Supplement1A:** w;20XUAS-6XEGFP; ETHRA-P65AD/CCAP-R^MI05804^-Gal4DBD

**Figure 6—Figure Supplement1B:** w;20XUAS-6XEGFP; ETHRB-P65AD/CCAP-R^MI05804^-Gal4DBD
