# Supplementary material for: Neuromodulatory connectivity defines the structure of a behavioral neural network
Source: eLife. 2017 Nov 22;6:e29797. doi: 10.7554/eLife.29797 (PMC5720592; doi:10.7554/eLife.29797)
Supplement: Supplementary file 2. [file elife-29797-supp2.docx]

**Supplementary File 2: Sequences of DNA Constructs Used to Make ETHRB-p65AD and**

**Burs-LexA::VP16AD Lines**

**Sequence of “ETHRB^MI00949^-p65AD in 4b” construct:**

atacattttttttctagacagaaatatactttaattcaactaaattaatatatatatgtacctctcccatcttttagctattaaatagtcgaggaactcgactatagtattatatccggttttggtttaaaaaccaatcattgtttttttttaacgacacagtactatttaatatgggcttcaacatatattttaaatgatgaacccaagaaatgaaaaaaccataaaaacaagcggtcatgaaaatcgcgtcctggccagactggtacattggaaacttgaacttcatcccagactgggttaaccttttgcccatcgccattctctcatcatcacaaacagcttctcttcctgcctgctgaaacctcttctttttcccgccactcttggccttttcctccaaaatccaaccgtatccattatccattatccacgcccccggtaaccccgccacctgattacgtttcttgtttgctttctctccccaatctgcaatatcccccgagcagccccattctgtgggtggccgagtacaagctggccgagtacattgatggatcgtcggtggccgtgtgcctgacccaggccatcagcgactggacgctggccttcttcctgatgaccatctcggtgttcttcgtggtgccgttcgtgaccctggtggtgctgtacggcatcatcgcccggaatctggtctccaacagggcggccatgctgcgcgcccgtcccacgaagccggagcttagtctgaaggcccgcaagcaggtggtcctaatgctcggcgccgttgtgctgtccttcttcgtctgcctgctgcccttccgcgtcctcaccctgtggatcatcctcagcacggaccaaacactgcacgatctgggattggtgcgctactacagcttgctgtacttctgtaggatcatgttgtacctcaactcggccatgaatccgattctttacaacctgatgtccaccaagtttcgaaggggtttcaagcggctttgtcaggacgcagggcgattactgctggaattggtgacattgggaagaaggaaggaagactcttctcgcgggcgcagaggtaccttgtcactgggcatgggcaccaatacgaacacgaataccaactcctcaaatgccacaggagccacgagctccagcattctctcgagaagctccaatcgcaggtgcagcgaggatatcagtcgcacccgccttaagatcgagatgcagatgccatgtggcagcgacctggaggccatggccatgctgcagcattccaccttgggcaagggaattgccaggcgagtgagtgatagccgtctcatgcctctgagaaatcatcaaccacgacgccacaagccgcaaataagtttcgacgaggagtcactggaggagaataagcgtagtgaagcgaaaatcccaacaaaatgtcgagaaaagcttcctggaatagcaagagaaatagtaaatctgaccgaaaataccctcggatccgcgagatgggcgtgaaaaatcgcctttaacccgtttacacactcgcttcacttggctggaatgtgccgatgacgctgatcccgatcccgattcctattcccgctcctggtaaacacaaatcgcttcctgaacgcttttggcagccataatcgatttcagtcacccgctgacagctgccgagaatcccgaatcgtagttgggctgccgccttggacatttccgtaccaaaaacaatcaatttttcgggctattaaactcgaatgaatgtgccaaagataaataacagcaacgacagccgaacagcactaaaactaccactactggtcagggttaggagtccagatatcgactggcagtaaggggtaagccattaggtggccttcgaggagcggagctggcaaatggaaatggcttaatatgcgtttgggcgaaccatcaggtcggcggataaacatgatttaggtgtattgaaatagtttccgtacgccggaccgtacaaaatggccgccattacacgggcggccatcaccggaagtcgaaaaaactgttgcctacttgccggcagctcaaataaaaagcttagctcaatatgcacacaatgtccacatgtaaacacagctgtacatacacatacatacccccacacgcacacatagccaaatattaatatttatatttgtacgaacatatttatatacacatatttacatatatatagtctgcttataaatatgtacaaggaaccacacgcacacactcacacacacacgagagtttgtaagtaaataaattttttatgacaactcatggcgggacttttccacattaccccatccaatcggaatctgtaatttatataattctaaggaaatcggacaaatgttcgtcctctgtaaatatacgtattcgtaccatgcgcgtagctttttaatttgttttcgtagccctatgacgtagactcccacttttgtagagtaaaaagtaataagtagcttgaacccacaccatcctgcgagtatgatgagtatgagcagacgctgccgtttccgtttccgctgtgtgattaagttgagtgcttcgcccccgaaacttttccagtagttagccgttgagtagcttttaaatatttgtttgtttcctgcccgcagcgcgataaaactttgcagacagcgctagtcactctagtccccgctaaaactgataatatgattcttgcagtcccatcatcgccatatccacctacagcgtggaaccctacggggacggaaccgatgctcccgtttgcaccaccgccgccgatggtttctggtcgatcttctacttcgtgggctgcatcacggtgtttttcttcctgcccttcggcatcctggttcttctatacgcggccatcgcttacaagctgctccgtcccaacaacgccttccaccgaccaacctccccgcagccacagcagccgtccggcggagccaccagtggctcctcacaggtgcccagcaccaagggtaacagccatcaacaaagcaacgggatgaggaagcatcgcaagcaggtaatcttcatgctggtggccgtggtgtctagcttttttgtttgccttctacccttccgggccttcaccctctgggtgatcctggccagcgccgaggatgtcgagggtctgggcattgccggctactacaacctgctgtacttctcgcgcttcatgctctacctaaactcagccatgaacccgatcctctacaacctgatgtcctccaaatttcgcagcggcttctggcggctgctgctcacttgtctgggccaacggccacatcaccaccatcgccaccactaccatcagaggcagcatccaacggcaggcggaagtgggcgcaatgcgtccacgcgacaggaacaggatgccgaggaaggagctgcgctggcgggaacgaccagcgcccgacatccacgtcgcacactccgccgcgaggccaccttcttgatcaactccatatccacctcctcgggtacggatcgcaccacatcatcatcggcgtggcgcagcaacagtctgtccatttccggtctgagcgaacgggagcgcggcatactgggagccgctatcatcggcacaacggctgccaccgttacaaccgcctgtctgcaggagcgacgcgccagcaagatcgagggccgcggcagcctgctgacctgcggcgatgtggaggagaaccccgggcccatggataaagcggaattaattcccgagcctccaaaaaagaagagaaaggtcgaattgggttccacgccgatggagttccagtacctgcccgatacggatgaccgtcaccgtatcgaagaaaagcggaagcgaacctatgaaaccttcaagtccatcatgaaaaagtcccccttctcgggccccacggacccgcgccccccgccccgtcgtattgcggttccttcgcgcagcagtgccagcgtccccaaacccgcaccgcagccctacccgttcacttcctcccttagcacgattaactatgatgagttccccacgatggtgttccccagtggacaaatttcccaggcatcggcactggctccggccccaccgcaagtcctcccccaggcgcccgctccggcaccggctcccgcaatggtgagtgctctggcccaggcccccgctccagtccccgtgctggcgcctggacccccacaggcagttgcccctcctgctccgaaaccaacgcaggcgggcgaaggaaccctgagcgaggccctcttgcagcttcagttcgatgacgaagacttgggagccctgctgggtaacagcacagaccctgccgtattcaccgatctcgcatccgtggacaacagcgagtttcagcagctcttgaatcagggaatcccggtcgcacctcataccacagagcccatgctgatggaatacccggaggctatcacgcgactggtgaccggcgcacagcgaccacccgatccagcccctgccccactgggtgccccgggtttgcccaatggcctcctcagcggcgatgaggatttctccagcatcgctgatatggatttctccgctttgctgagccagataagctccgctagcggaggaggtggtggaggtggaggtggaggtactagtctggagatcgaggccgccttcctggagcgcgagaacaccgccctggagacccgcgtggccgagctgcgccagcgcgtgcagcgcctgcgcaaccgcgtgagccagtaccgcacccgctacggccccctgggcggcggcaagtaaagatctgaatcccatcctaaatatgcatccttcgagtccctgtccactgccagcaaccgttaaagtatttagttgaattccaacagcaaatagtcatagttttagaaaaattttgttgtaaagactgaagtacctcagaagaagtcaataaacgcaagagaagatgttttttaaaatgaatcgtagatactgaaaaaccccgcaagttcacttcaactgtgcatcgtgcaccatctcaatttctttcatttatacatcgttttgccttcttttatgtaactatactcctctaagtttcaatcttggccatgtaacctctgatctatagaattttttaaatgactagaattaatgcccatcttttttttggacctaaattcttcatgaaaatatattacgagggcttattcagaagcttatcgataccgtcgaataaagccaaatagaaattattcagttctggcttaagtttttaaaagtgatattatttatttggttgtaaccaaccaaaagaatgtaaataactaatacataattatgttagttttaagttagcaacaaattgattttagctatattagctacttggttaataaatagaatatatttatttaaagataattcgtttttattgtcagggagtgagtttgcttaaaaactcgttt

**Sequence of drosophilized LexA::VP16AD DNA construct:**

atgaaggccctgaccgcccgccagcaggaggtgttcgatctgatccgcgatcacatcagccagaccggcatgccccccacccgcgccgagatcgcccagcgcctgggcttccgcagccccaacgccgccgaggagcacctgaaggccctggcccgcaagggcgtgatcgagatcgtgagcggcgccagccgcggcatccgcctgctgcaggaggaggaggagggcctgcccctggtgggccgcgtggccgccggcgagcccctgctggcccagcagcacatcgagggccactaccaggtggatcccagcctgttcaagcccaacgccgatttcctgctgcgcgtgagcggcatgagcatgaaggatatcggcatcatggatggcgatctgctggccgtgcacaagacccaggatgtgcgcaacggccaggtggtggtggcccgcatcgatgatgaggtgaccgtgaagcgcctgaagaagcagggcaacaaggtggagctgctgcccgagaacagcgagttcaagcccatcgtggtggatctgcgccagcagagcttcaccatcgagggcctggccgtgggcgtgatccgcaacggcgattggctggaattccccgggatccgccgccccgccggcatccccggggatctggccccccccaccgatgtgagcctgggcgatgagctgcacctggatggcgaggatgtggccatggcccacgccgatgccctggatgatttcgatctggatatgctgggcgatggcgatagccccggccccggcttcaccccccacgatagcgccccctacggcgccctggatatggccgatttcgagttcgagcagatgttcaccgatgccctgggcatcgatgagtacggcggctaa
